# Development of a spatially indexed dose–volume histogram for treatment plan evaluation

**DOI:** 10.1093/jrr/rrag050

**Published:** 2026-07-15

**Authors:** Hideharu Miura, Shuichi Ozawa, Masahiro Kenjo

**Affiliations:** Hiroshima High-Precision Radiotherapy Cancer Center, 3-2-2, Futabanosato, Higashi-ku Hiroshima 732-0057, Japan; Department of Radiation Oncology, Institute of Biomedical & Health Sciences, Hiroshima University, 1-2-3 Kasumi Minami-ku Hiroshima-shi, Hiroshima 734-8553, Japan; Hiroshima High-Precision Radiotherapy Cancer Center, 3-2-2, Futabanosato, Higashi-ku Hiroshima 732-0057, Japan; Department of Radiation Oncology, Institute of Biomedical & Health Sciences, Hiroshima University, 1-2-3 Kasumi Minami-ku Hiroshima-shi, Hiroshima 734-8553, Japan; Hiroshima High-Precision Radiotherapy Cancer Center, 3-2-2, Futabanosato, Higashi-ku Hiroshima 732-0057, Japan

**Keywords:** spatially indexed dose–volume histogram, spatially informed plan evaluation, laryngeal cancer, carotid artery sparing, volumetric-modulated arc therapy

## Abstract

Conventional dose–volume histograms (DVHs) lack spatial information, a critical limitation in evaluating carotid artery sparing in laryngeal volumetric-modulated arc therapy (VMAT). We developed a spatially indexed DVH that integrates the distance between the planning target volume (PTV) and carotid arteries for a more comprehensive plan quality assessment. Three representative cases (distant, intermediate, proximal) were analyzed. For each carotid voxel, dose and minimum Euclidean distance to the PTV were calculated. A color-coded cumulative DVH was generated by mapping the mean dose-specific distance (*MD*_D_) to a color gradient (red: proximal, blue: distal). Spatial metrics at 35 Gy were derived: minimum distance (*d*_min,35Gy_), mean dose-specific distance (*MD*_35Gy_) and *V*_35Gy,10mm_ (volume ≥35 Gy within 10 mm of PTV surface). The spatially indexed DVH visually identified spatial risk transitions. In the distant case, *V*_35Gy,10mm_ was negligible (0.1 cc and N/A). In the intermediate case, despite a low conventional *V*_35Gy_ (0.9 and 1.8 cc), proximity metrics revealed high-risk areas with a *d*_min,35Gy_ of 3.2 mm and *V*_35Gy,10mm_ of 0.8 and 1.4 cc. The proximal case showed the highest risk, with the DVH shifting to red above 30 Gy; nearly all *V*_35Gy_ (2.4 and 2.3 cc) was within the 10-mm high-risk zone (*V*_35Gy,10mm_: 2.0 and 1.7 cc), with a *d*_min,35Gy_ of 2.2 and 1.4 mm. The spatially indexed DVH provides an intuitive method to evaluate the spatial relationship between high-dose regions and the PTV, facilitating better risk stratification and optimization in laryngeal VMAT.

## INTRODUCTION

Dose–volume histograms (DVHs) are fundamental tools for evaluating radiation treatment plans, providing a concise summary of three-dimensional dose distributions within target volumes and surrounding organs at risk (OARs) [1]. Despite their widespread use, conventional DVHs lack spatial information, offering no indication of where doses are distributed within anatomical structures. This limitation becomes particularly important in clinical scenarios where spatial dose distribution directly affects the risk of toxicity. The clinical importance of spatial dose information has been demonstrated at several treatment sites. In prostate radiotherapy, the proximity of the anterior rectal wall and bladder neck to the planning target volume (PTV) is associated with late gastrointestinal and genitourinary complications [2, 3]. Similarly, in head and neck cancer treatment, sparing the superior regions of the parotid glands has been linked to improved recovery from xerostomia [4]. These examples highlight the need for spatial dose assessment methods to optimize treatment planning and better evaluate toxicity that conventional DVHs fail to represent.

Radiation therapy is also the standard organ-preserving treatment for early- and intermediate-stage laryngeal cancer, offering excellent local control and functional preservation [5–7]. While volumetric-modulated arc therapy (VMAT) enhances dose conformity to the target [8–11], clinically significant late toxicities remain a major concern for long-term survivors [12–16]. These toxicities are most notably represented by carotid artery stenosis and subsequent cerebrovascular events. Carotid sparing is inherently challenging due to variations in patient-specific anatomy, as the spatial relationship between the PTV and carotid arteries can range from direct contact to separation by a soft-tissue layer. Conventional DVHs cannot represent such spatial relationships, even when different treatment plans appear equivalent in their dose–volume statistics. Consequently, an evaluation framework that incorporates the spatial proximity between the PTV and OARs is needed to better characterize plan quality.

The primary objective of this proof-of-concept study was therefore to develop a spatially indexed DVH that integrates the distance between the PTV and OARs into conventional DVH analysis. As a representative clinical scenario in which trade-offs between the PTV and OARs are particularly pronounced, we focused on laryngeal VMAT and carotid artery sparing.

## MATERIALS AND METHODS

### Patient selection

This retrospective dosimetric study analyzed a cohort of patients with early- or intermediate-stage laryngeal cancer who had previously undergone VMAT at our institution. To demonstrate the performance of the proposed spatially indexed DVH across various anatomical configurations, three representative cases (Cases 1–3) were selected to cover the range of PTV–carotid arteries distance typically encountered in clinical practice. The three representative cases were classified according to the minimum PTV–carotid distance: the proximal case had a minimum distance <3 mm on at least one side, the intermediate case had minimum distances between 3 and 8 mm bilaterally and the distant case had a minimum distance ≥8 mm on both sides. This study was conducted in accordance with the Declaration of Helsinki and was approved by our Institutional Review Board.

### Treatment planning

All patients were scanned in the supine position using a dedicated thermoplastic head-and-neck mask for immobilization. Planning computed tomography (CT) images were acquired with a multi-slice CT scanner (Discovery CT580 RT; GE Healthcare, Chicago, IL) at 120 kV, with a 2.5-mm slice thickness. Patients were instructed to refrain from swallowing during image acquisition to ensure a stable laryngeal position, and the same instruction was consistently given during treatment delivery. The clinical target volume (CTV) was delineated by an experienced radiation oncologist, typically encompassing the larynx from the hyoid bone to the cricoid cartilage.

The PTV was generated by applying an anisotropic margin to the CTV: 3 mm posteriorly and 5 mm in all other directions. The reduced posterior margin was specifically designed to spare the esophagus and carotid arteries. The common and internal carotid arteries, along with the spinal cord, were contoured as OARs. These contours were uniformly extended 1 cm cranially and caudally beyond the PTV to ensure a sufficient assessment of dose fall-off.

**Fig. 1:**
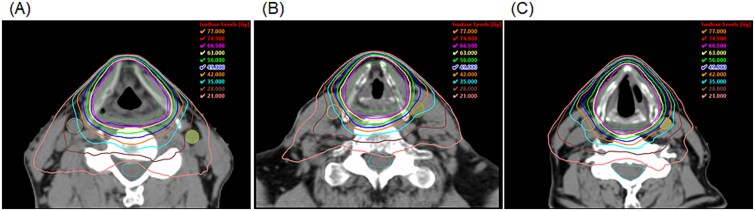
Dose distributions for the three representative cases. Axial CT images showing the PTV and carotid artery contours with isodose lines for (**A**) distant, (**B**) intermediate and (**C**) proximal cases.

VMAT plans were generated using the Eclipse treatment planning system (Varian Medical Systems, Palo Alto, CA) for 6-MV photons delivered by a TrueBeam STx linear accelerator. For this methodological comparison, all three plans were generated using the same single-arc VMAT geometry (gantry rotation range 140°–220°) and a 10° collimator angle. The prescription dose and optimization priorities were identical across cases, with PTV coverage, carotid sparing and spinal cord protection as the main objectives. The relative weighting among these objectives was adjusted according to the PTV size and the spatial relationship between the PTV and carotid arteries. Dose distributions were calculated using the Acuros XB algorithm with a 1.0-mm grid size, consistent with our routine clinical setting for laryngeal VMAT, to accurately calculate dose distributions in regions with steep dose gradients around the carotid arteries. The prescribed dose was 70 Gy in 35 fractions, normalized to the PTV median dose (D_50%_). Figure 1 displays representative dose distributions for each anatomical category.

### Spatially indexed DVH

**Fig. 2:**
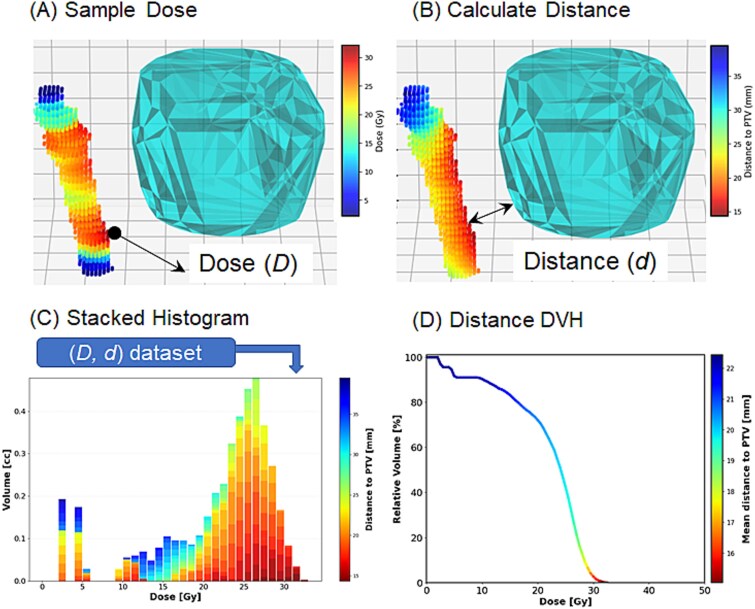
Workflow of the spatially indexed DVH. (**A**) Voxelization and dose sampling (*Di*) of the carotid artery. (**B**) Calculation of the minimum Euclidean distance (*di*) to the PTV surface. (**C**) Stacked differential DVH, where each bar represents volume partitioned by distance to the PTV. (**D**) Cumulative DVH with a continuous gradient representing the mean dose-specific distance from the PTV.

To integrate spatial information into the dosimetric analysis, we developed a computational method to correlate voxel-specific dose with its anatomical location relative to the target, as illustrated in Fig. 2. Throughout this manuscript, we use uppercase *D* to denote dose and lowercase *d* to denote the Euclidean distance from the PTV surface. First, three-dimensional binary masks for the PTV and carotid arteries were extracted from the DICOM-RT structure set and resampled to match the 1.0-mm isotropic dose grid. For each voxel *i* within the carotid artery volume, the absorbed dose *D_i_* was determined using trilinear interpolation, and the corresponding spatial metric *d_i_* was defined as the minimum Euclidean distance to the nearest PTV surface voxel. In cases where an OAR voxel is located inside the PTV, the minimum distance to the PTV surface becomes zero by definition, and such voxels are assigned *d_i_* = 0 mm and included in the calculation of all distance-based indices. This dual-parameter extraction yielded a dataset of (*D_i_, d_i_*) pairs for the entire organ volume.

Unlike conventional differential DVHs, our approach introduces a secondary partitioning of each dose bin according to predefined distance intervals. In the resulting stacked histogram, the height of each segment represents the partial volume at a specific distance, providing a quantitative visualization of dose localization. This representation enables a clear distinction between high-dose regions physically adjacent to the PTV and those located distally, which may be associated with different clinical risks.

To translate these spatial data into a clinically intuitive format, we developed a color-coded cumulative DVH that supplements conventional DVH curves with proximity information. For any given dose threshold *D*, the mean dose-specific distance (*MD_D_*) was calculated by averaging the Euclidean distances *d_i_* of all carotid voxels receiving a dose ≥ *D*. We used the simple arithmetic mean without dose weighting to describe the geometric localization of all voxels exceeding the threshold, irrespective of local dose variations. This scalar value *MD_D_* was then mapped to a continuous color-gradient scale, ranging from red (proximal) to blue (distal), and applied to the corresponding segment of the cumulative DVH curve. This integrated visualization allows simultaneous evaluation of conventional dose–volume relationships and the physical localization of dose within a single interface, facilitating the identification of cases where high-dose regions are adjacent to the PTV surface.

### Quantitative analysis

To evaluate the added value of spatial information, both conventional and spatially indexed metrics were calculated for each plan. First, conventional volumetric parameters (*V_D_*) were calculated as a baseline. We specifically selected *V*_35Gy_ as the primary conventional metric, because 35 Gy represents an intermediate dose level corresponding to ~50% of the prescription dose (70 Gy) and thus a readily identifiable isodose level during treatment planning. Intermediate carotid doses in this range have been used in previous carotid dosimetry studies (e.g. *V*_35Gy_, *V*_50Gy_ and *V*_63Gy_) [15, 16] and have been implicated in radiation-induced carotid wall changes and stenosis, although no single universally accepted dose cut-off has been established. This threshold can be modified as needed according to institutional practice or organ-specific considerations.

1. Minimum distance at 35 Gy (*d*_min,35Gy_): The shortest Euclidean distance from the PTV surface to any carotid voxel receiving at least 35 Gy. This index identifies the point of closest proximity between the high-dose region and the target.
2. Mean dose-specific distance (*MD*_35Gy_): The average distance to the PTV for all carotid voxels receiving ≥35 Gy. Unlike *d*_min,35Gy_, this metric provides a global assessment of the spatial concentration of dose within the organ.
3. *V*_35Gy,10mm_: The absolute volume receiving ≥35 Gy that is simultaneously located within ≤10 mm of the PTV surface. This dual-constraint metric was designed to isolate the most clinically concerning portion of the OAR, where high dose and high proximity overlap.

The distance threshold of 10 mm was selected as a practical example of a high-risk proximity zone in our laryngeal VMAT setting rather than as a strict clinical cutoff. This value can be adjusted, and the same spatial metrics can be recalculated for alternative distance thresholds as appropriate for different organs or planning objectives. In cases where no carotid voxels received a dose ≥35 Gy (i.e. *V*_35Gy_ = 0 cc), *d*_min,35Gy_ and *MD*_35Gy_ were not computed and are reported as ‘N/A’ throughout the manuscript.

## RESULTS

### Visualization of spatially indexed DVH

**Fig. 3:**
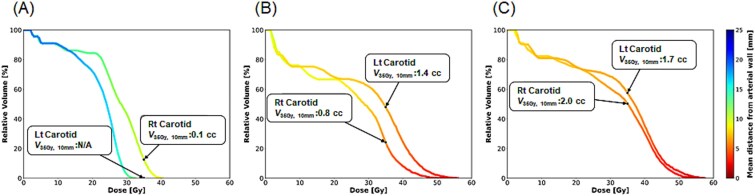
Spatially indexed cumulative DVHs. Color-coded curves show the mean distance between the PTV and the carotid arteries at each dose level for the (**A**) distant, (**B**) intermediate and (**C**) proximal cases, with lower values indicating closer proximity to the PTV and higher values indicating greater distances.

The spatially indexed cumulative DVHs integrated spatial proximity into the conventional DVH for all three anatomical configurations, as shown in Fig. 3. In the distant case, the DVH curves were characterized by cooler colors (blue to cyan), indicating that even the regions receiving intermediate doses were located more than 20 mm from the PTV surface. Conversely, as the PTV–carotid arteries separation decreased in the intermediate and proximal cases, a progressive color shift toward warmer tones (yellow to red) was observed. Specifically, the proximal case displayed a prominent transition to red at dose levels exceeding 30 Gy, visually indicating that the highest dose components were concentrated within 10 mm of the PTV surface.

### Performance of distance-based metrics

The spatial dose metrics at 35 Gy for the distant, intermediate, and proximal cases are summarized in Table 1.

**Table 1:** Geometric relationship between the PTV and carotid arteries (prescription: 70 Gy in 35 fractions)

Category	*V* _35Gy_ (cc)	*d* _min,35Gy_ (mm)	*MD* _35Gy_ (mm)	*V* _35Gy, 10mm_ (cc)
Distant	0.6 / 0.0	8.4 / N/A	17.4 / N/A	0.1 / N/A
Intermediate	0.9 / 1.8	3.2 / 3.2	7.3 / 8.4	0.8 / 1.4
Proximal	2.4 / 2.3	2.2 / 1.4	7.5 / 7.9	2.0 / 1.7

Values are presented as right/left carotid artery. PTV = planning target volume, *V*_35Gy_ (cc) = volume of the carotid artery receiving at least 35 Gy, *d*_min,35Gy_ = minimum distance from the PTV to the 35 Gy isodose surface within the carotid artery, *MD*_35Gy_ = mean distance between the PTV and the carotid artery at the 35 Gy dose level, *V*_35Gy,10mm_ = volume receiving ≥35 Gy within 10 mm of the PTV surface. *d*_min,35Gy_ and *MD*_35Gy_ are reported as NA when no carotid voxels receive at least 35 Gy.

In the distant case, the conventional *V*_35Gy_ was minimal (0.6 and 0.0 cc for the right and left carotid arteries, respectively). Spatial metrics also indicated a low-risk profile, with *d*_min,35Gy_ of 8.4 mm on the right side and no voxels receiving ≥35 Gy on the left side, resulting in an *MD*_35Gy_ of 17.4 mm and a *V*_35Gy,10mm_ of only 0.1 cc on the right and N/A on the left.

In the intermediate case, *V*_35Gy_ increased modestly to 0.9 and 1.8 cc for the right and left carotid arteries, respectively, compared with the distant case. However, proximity metrics showed a pronounced shift toward the PTV, with *d*_min,35Gy_ of 3.2 mm on both sides and *MD*_35Gy_ of 7.3 and 8.4 mm. Consistent with this, *V*_35Gy,10mm_ increased to 0.8 and 1.4 cc, indicating that a larger portion of the irradiated volume was positioned close to the PTV.

The proximal case demonstrated the highest spatial risk. Compared with the intermediate case, *V*_35Gy_ further increased to 2.4 and 2.3 cc, while *d*_min,35Gy_ decreased to 2.2 and 1.4 mm and *MD*_35Gy_ to 7.5 and 7.9 mm for the right and left sides, respectively. Consequently, *V*_35Gy,10mm_ reached 2.0 and 1.7 cc, confirming that nearly all of the volume receiving 35 Gy was located within the high-risk 10-mm zone.

## DISCUSSION

This study demonstrated the feasibility and potential of the spatially indexed DVH as a tool for spatially informed plan evaluation in laryngeal VMAT. Our proposed method integrates the anatomical relationship between the PTV and the carotid arteries, providing critical spatial information that is inherently missing from conventional DVH analysis. By incorporating distance as a secondary parameter, this approach allows for a more detailed characterization of plan quality and risk stratification beyond simple volume-based metrics. Our findings suggest that the spatially indexed DVH facilitates the distinction between plans with similar dose-volume profiles by using color-coded proximity mapping and the *V*_35Gy,10mm_ metric to reveal distinct spatial risk patterns. For instance, even when *V*_35Gy_ values were relatively similar between cases, the proposed method identified a marked escalation in spatial risk as the minimum distance at 35 Gy decreased and the *V*_35Gy,10mm_ increased accordingly. By explicitly integrating the PTV–OAR distance into the evaluation process, clinicians can distinguish unavoidable high-dose exposure driven by target proximity from dose that may still be reduced through further optimization. In practice, when distance-colored DVH segments above a clinically relevant threshold are predominantly represented by warm colors, this indicates that most high-dose voxels lie close to the PTV surface and that additional sparing is likely constrained by anatomy. Conversely, if a substantial portion of the high-dose region appears in cooler colors at larger distances, the plan may contain avoidable dose spill to distal parts of the organ, where modifications to arc geometry or OAR weighting could reduce dose without compromising target coverage. Taken together, these patterns provide clinicians with an intuitive framework for interpreting the spatially indexed DVH in daily practice.

Integrating spatial information into dose-volume metrics remains a central challenge in radiotherapy plan evaluation [2–4, 17, 18]. Although previous approaches, such as z-dependent DVHs (zDVH) and dose-surface histograms (DSHs), have attempted to bridge this gap, they often lack the intuitive simplicity and visual immediacy required for routine clinical implementation. For instance, while zDVHs provide slice-by-slice volumetric data, they do not explicitly characterize the three-dimensional proximity between the PTV and OARs [17]. Similarly, while DSHs and surface map features can effectively characterize the dose distribution on an organ’s surface, they do not explicitly incorporate the distance from the target as a primary evaluation dimension [2]. By contrast, the proposed spatially indexed DVH augments the familiar cumulative format with color-coded proximity information, enabling clinicians to simultaneously identify high-dose regions and their spatial relationship to the target surface. This intuitive visualization is especially valuable in laryngeal VMAT, where the potential for carotid sparing is highly dependent on individual patient anatomy [9, 10]. Although this proof-of-concept study focused on the carotid arteries in laryngeal cancer, the spatially indexed DVH framework is applicable to other serial OARs in which localized high-dose regions near the target are critical for toxicity (e.g. optic pathways, spinal cord, proximal bronchi). For predominantly parallel organs such as the parotid glands or lungs, conventional dose–volume metrics may already summarize most of the relevant information. However, in selected scenarios, such as when assessing dose relative to functional subregions, integrating PTV–OAR distance into DVH analysis could still offer additional insight.

Despite its advantages, this study has several limitations. First, as a proof-of-concept study, our analysis was limited to a small number of illustrative cases to focus on the methodological framework. While these cases effectively demonstrated the utility of the spatially indexed DVH, a larger cohort is necessary to establish robust clinical thresholds for the proposed spatial metrics. Second, while the spatially indexed DVH provides a more spatially explicit representation of dose distribution, it remains a dosimetric evaluation tool rather than a direct predictor of biological injury. The clinical significance of metrics such as *V*_35Gy,10mm_ in predicting actual vascular complications or late cerebrovascular events must be further validated through prospective clinical correlation.

In conclusion, we developed the spatially indexed DVH framework that integrates spatial proximity into conventional dosimetric evaluation for laryngeal VMAT. By utilizing distance-based metrics, this approach provides a more detailed and intuitive characterization of plan quality than conventional DVHs.
